# Commentary: New Insights in Anorexia Nervosa

**DOI:** 10.3389/fnins.2016.00483

**Published:** 2016-10-25

**Authors:** Per Södersten, Cecilia Bergh, Michael Leon

**Affiliations:** Section of Applied Neuroendocrinology, Mandometer Clinic, Karolinska InstitutetHuddinge, Sweden

**Keywords:** anorexia nervosa, dopamine, ghrelin, physiology, treatment

In a comprehensive overview of the concomitants of *anorexia nervosa* (AN), Dr. Gorwood and collaborators list nine hormones “driving of the reward aspect of thinness” and “abnormal satiety feedback,” and they incorporate new neuropeptides, sociocultural, psychological, psychiatric co-morbidity, genetic, epigenetic and gut microbiological factors and 11 risk factors into a “holistic model of AN,” including “body, brain and mind”.

We regard this list of potential mechanisms underlying AN as introducing redundant complexity to an area of study that continually mistakes the *causes* of AN with the responses to starvation that are the *consequences* of the disorder (Södersten et al., 2008, 2014, 2016; Zandian et al., 2015). In particular, we wish to comment on two of the topics that the authors discuss: the response of the mesolimbic dopamine neurons to a reduction in the availability of food and the change in the levels of ghrelin that is associated with AN.

## Dopamine and starvation

To meet the challenge of starvation, animals have developed efficient foraging strategies that are mediated by dopamine (Stephens and Krebs, 1986; Faure et al., 2008; O'Connell and Hofmann, 2011; Richard and Berridge, 2011; McCue, 2012; Bédécarrats et al., 2013; Moe et al., 2014; Søvik et al., 2014; Masek et al., 2015). Moreover, hunger hormones influence how animals relate to environmental food cues by acting on dopamine neurons (Cone et al., 2015). For example, dopamine is involved in evaluating the hedonic aspects of food (Berridge and Kringelbach, 2015) and increases in response to the initial food restriction in patients who become anorexic, thereby encouraging the continuation of such behavior (Södersten et al., 2008). Indeed, such neural changes may have been necessary to allow some individuals in the population to withstand famine, the major challenge to humans on this planet until recent times. Therefore, changes in the responses of the mesolimbic dopamine terminals in the ventral striatum of AN patients, which Dr. Gorwood and collaborators discuss, should be understood as the normal physiological response to starvation (Södersten et al., 2016).

We proposed that increased mesolimbic dopamine activity initially supports food restriction, and as food restriction results in severe weight loss, it precipitates the other features of the disorder (Södersten et al., 2008; Ioakimidis et al., 2011).

## Ghrelin and starvation

Because AN patients eat very little food, Dr. Gorwood and collaborators find the increase in the level of ghrelin, an “orexigen,” in AN “paradoxical” and they suggest that anorexics have a “global resistance to orexigenic signals.” However, this conclusion is inappropriate, just as the reconsideration of the “neuropeptide tyrosine (NPY) paradox” has demonstrated. Thus, whereas sedentary rats eat more food when treated with NPY, they increase their foraging for food, but eat less when food availability is restricted (Södersten et al., 2008). An “orexigenic” hormone, NPY, turns into an “anorexigen” when the food environment changes from abundance to scarcity, questioning the usefulness of the concept of orexigens and anorexigens (Zandian et al., 2015). Similarly, the older concepts of excitation and inhibition exerted by anatomically separable hypothalamic centers that were thought to maintain body weight homeostasis (Stellar, 1954) also seem not to be applicable to the normal control of food intake (Södersten et al., 2008).

In the same way, ghrelin stimulates foraging when the supply of food is continuously limited, but it *stimulates* eating when food is temporarily removed (Méquinion et al., 2015). Far from being “paradoxical” or even “inappropriate,” increased physical activity associated with low leptin levels and high plasma concentrations of ghrelin are adaptations to a long-term reduction in food intake by encouraging the production of heat to maintain thermal homeostasis in individuals whose surface area remains the same, but whose body mass that produces heat has greatly diminished. This response is therefore beneficial, rather than pathological in experimental animals or AN (Méquinion et al., 2015). These changes in ghrelin may be a response to low body weight and low food intake, as constitutionally thin women also have high levels of ghrelin (Tolle et al., 2003). In addition, the hypothalamic neurons of mice, previously thought to be sensors of metabolic status, respond immediately to the introduction of food into their environment, even when pre-treated with ghrelin, supporting the idea that ghrelin does not actually control food intake (Chen et al., 2015).

## Effective treatment for anorexia nervosa

We have suggested that treatment of AN should focus on the disordered eating behavior, which is the major behavioral marker of AN, and that the other aspects of the anorexic phenotype emerge as a consequence of starvation and that they will also return to normal when eating behavior and food intake have been normalized (Bergh and Södersten, 1996). Indeed, in a randomized clinical trial, we showed that such a therapy is effective for AN (Bergh et al., 2002). Moreover, the outcome of this treatment for 1428 patients, including all eating disorders diagnoses, had a remission rate of 75%, with a rate of relapse of 10% over 5 years of follow-up, and no patient has died in treatment or follow-up (Bergh et al., 2013).

In stark contrast, as Dr. Gorwood and collaborators pointed out, standard treatments have very limited effect on AN, which is therefore associated with “the highest mortality rate of all psychiatric disorders …and exceptionally high relapse rates.” We therefore think that considering the complexities of the interactions between AN and starvation, while interesting, will be unlikely to lead to an even more effective treatment for this disorder.

## Author contributions

All authors contributed to this work and approved it for publication.

## Funding

This work was supported by Mando Group AB.

### Conflict of interest statement

PS and CB own 47.5% each and ML owns 5% in the stock of Mando Group AB, the company that treats patients with eating disorders through contractual agreements with the City Council of Stockholm. All health care, provided by public as well as private clinics, is publically funded by such contracts in Sweden.
